# Renewable, Eugenol—Modified Polystyrene Layer for Liquid Crystal Orientation

**DOI:** 10.3390/polym10020201

**Published:** 2018-02-17

**Authors:** Changha Ju, Taehyung Kim, Hyo Kang

**Affiliations:** Department of Chemical Engineering, Dong-A University, 37 Nakdong-Daero 550beon-gil, Saha-gu, Busan 604-714, Korea; 1771088@donga.ac.kr (C.J.); xogud1290@donga.ac.kr (T.K.)

**Keywords:** liquid crystal, orientation, polystyrene, eugenol

## Abstract

We synthesized a series of plant-based and renewable, eugenol-modified polystyrene (PEUG#) (# = 20, 40, 60, 80, and 100, in which # is the molar content of the eugenol moiety in the side group). Eugenol is extracted from clove oil. We used polymer modification reactions to determine the liquid crystal (LC) orientation properties of the polymer films. In general, the LC cells fabricated using the polymer films with a higher molar content of eugenol side groups exhibited vertical LC orientation behavior. The vertical orientation behavior was well correlated with the surface energy value of the polymer films. The vertical LC orientation could be formed due to the low polar surface energy value on the polymer film generated by the nonpolar carbon group. Electro-optical performances (e.g., voltage holding ratio (VHR), residual DC voltage (R-DC), and thermal orientation stabilities) were good enough to be observed for LC cells using PEUG100 polymer as an eco-friendly LC orientation material.

## 1. Introduction

Liquid crystal (LC) molecules have been intensively studied due to their attractive characteristics such as liquid-like fluidity and solid-like ordering [1]. LC molecules have been recognized to have anisotropic physico-chemical properties such as optical anisotropy and dielectric anisotropy induced by external stimuli due to their unique chemical structures [2]. Therefore, LC molecules have been applied for diverse fields, such as information technology [3,4,5], nanotechnology [6], biotechnology [7], and energy & environment technology [8,9] using interesting physico-chemical properties. For example, LC molecules have been widely utilized in the display industry, such as the transmissive one using nematic LC and the reflective one using cholesteric LC, respectively [10]. It is important to orientate the LC molecules that have anisotropy on the substrate in the same direction [10].

Orientation methods for liquid crystal molecules have been the focus of intensive development because of scientific and technical interest in flexible displays, as well as rigid panel displays [11,12,13,14,15,16]. The conventional method of mechanical rubbing of polyimide (PI) surfaces commonly used in the display industry produces stable LC orientation layers on these surfaces [17,18,19,20,21,22,23,24,25]. Polyimide derivatives containing hydrophobic long alkyloxy and/or alkyl side chains such as (*n*-decyloxy)biphenyloxy and *n*-octadecyl groups have been improved for use as vertical LC orientation layers [26,27,28,29]. However, PI derivatives require a high baking temperature of over 200 °C for their synthesis from the PI precursor to apply the LC orientation layers on plastic substrates for versatile displays. Therefore, new polystyrene (PS) derivatives containing long alkyl or fluoroalkyl chains, which are one of the comb-like polymers and an alternative to conventional polyimide derivatives, have been improved via a simple polymer reaction to produce vertical LC orientation layers for next-generation displays, including flexible displays, due to their advantages such as low temperature processability. For instance, LC cells fabricated using polymer films of *n*-alkylthiomethyl- and *n*-alkylsulfonylmethyl-modified polystyrenes that contain long alkyl groups (number of alkylcarbon > 8) exhibit vertical LC orientation characteristics [30]. LC cells made using 4-alkylphenoxymethyl-modified polystyrenes also show vertical LC orientation, even at a very high rubbing strength and irrespective of the length of the alkyl side groups [31].

Eugenol (2-methoxy-4-(prop-2-en-1-yl)phenol) is the major component (80–95%) of cloves [32]. It has been used extensively in perfumes, flavorings, and essential oils for a long time [33,34]. Eugenol has a methoxyphenol structure with a short hydrocarbon chain. In this structure, the hydroxy group of the aromatic ring in the eugenol is known to have antioxidant and antimicrobial properties [35,36,37]. For example, eugenol moieties inhibit the growth of pathogenic bacteria, as previously reported by other research groups [38,39,40,41,42]. Therefore, eugenol has been used in many fields and for many applications such as dental materials [43], inflammation medicines [33,44], and antioxidants in the plastics and rubber industries [45]. Eugenol can also be used for modifying the surfaces of substrates such as metal and glass using the hydroxy group via primary and secondary bonding for various film applications. The wettability on the surface of the modified film can be controlled by changing the eugenol content [46,47].

In this paper, bio-renewable eugenol modified PSs (PEUG#) were synthesized to inform vertical orientation of the LCs and investigate the effect of the molar ratio of the modified side groups on the LC orientation behavior. The optical and electrical characteristics of the LC cells made by using PEUG# films were also determined.

## 2. Materials and Methods

### 2.1. Materials

4-Chloromethylstyrene (CMS), eugenol, and potassium carbonate were commercially supplied by Aldrich Chemical Co. (Yongin, Korea). MLC-6608 (*n_o_*, *n_e_*, and Δ*ε* indicate ordinary refractive index, extraordinary refractive index, and dielectric anisotropy, respectively, *n_o_* = 1.4756, *n_e_* = 1.5586, and Δ*ε* = −4.2); nematic LC material, obtained in Merck Co. (Pyeongtaek, Korea). Tetrahydrofuran (THF), was dried using reflux method with sodium and benzophenone. Methanol and *N,N′*-dimethylacetamide (DMAc) were dried using molecular sieves (4 Å). 4-Chloromethylstyrene was purified using column chromatography filled with silica gel in eluting reagent, hexane, to eliminate nitroparaffin and *tert*-butylcatechol inhibitors and impurities. Poly(4-chloromethylstyrene) (PCMS) (*M_n_* and *M_w_*/*M_n_* indicate number average molecular weight and polydispersity index, respectively, *M_n_* = 30,000 and *M_w_*/*M_n_* = 2.10) was synthesized by free radical polymerization of 4-chloromethylstyrene using initiator, 2,2′-azoisobutyronitrile (AIBN), under a nitrogen atmosphere. AIBN was puchsed from Junsei Chemical Co., Ltd. (Tokyo, Japan) and purified using methanol by crystallization. Other reagents and solvents were used, as received.

### 2.2. Synthesis of Eugenol Modified Polystyrenes

The following procedure was used to synthesize all the PEUG#, in which # represents the molar content (%) of the eugenol moiety in the side group as previously reported in a similar procedure [48]. The synthesis of the PEUG100 is given as an example. Eugenol (1.61 g, 9.83 mmol, 150 mol % compared to poly(4-chloromethylstyrene)) and potassium carbonate (1.631 g, 11.81 mmol) mixture in *N,N′*-dimethylacetamide (30 mL) was heated up to 75 °C. Poly(4-chloromethylstyrene) (1 g, 6.56 mmol) dissolved in *N,N′*-dimethylacetamide (20 mL) was added to eugenol and potassium carbonate mixture and then stirred using magnetic bar in a nitrogen atmosphere at 70 °C for 24 h. The solution was cooled down to room temperature and poured slowly into methanol to yield white precipitate. The precipitate was further purified and washed with methanol to eliminate remaining *N,N′*-dimethylacetamide, potassium carbonate, and other used materials. The PEUG100 was dried in vacuum overnight to obtain a yield of over 80%. The substitution ratio of chloromethyl to eugenyl methyl ether was confirmed to be close to 100% within experimental error.

PEUG100 ^1^H NMR (CDCl_3_): δ = 0.8–1.9 (3H), 3.1–3.2 (2H), 3.7–3.8 (3H), 4.7–5.0 (2H), 5.0–5.9 (2H), 5.8–6.0 (1H), 6.3–7.2 (7H).

The other polystyrene derivatives containing eugenol side groups were synthesized using the same procedure as for the preparation of PEUG100 except for differing amounts of eugenol in the reaction. For example, PEUG80, PEUG60, PEUG40, and PEUG20 were prepared with eugenol amounts of 0.86 g (5.21 mmol), 0.64 g (3.92 mmol), 0.43 g (2.59 mmol), and 0.21 g (1.30 mmol), respectively, using slightly larger amounts of potassium carbonate (1.631 g, 11.81 mmol, 180 mol % compared with poly(4-chloromethylstyrene)).

### 2.3. Film Preparation and LC Cell Assembly

Solutions of PEUG# in tetrahydrofuran (1 wt %) were prepared and filtered using a polytetrafluoroethylene (PTFE) membrane with a pore size of 0.45 μm. Thin films of the polymers were spin-coated on glass substrates at 2000 rpm for 60 s. The LC cells were produced using the polymer films on glass slides and the films facing each other using spacers with thicknesses of 4.25 μm. Subsequently, the cells were filled with the nematic LC material MLC-6608 and were sealed with epoxy glue.

### 2.4. Instrumentation

The ^1^H-nuclear magnetic resonance (NMR) spectroscopy measurements were performed on Bruker AVANCE spectrometer (Bruker Co., Billerica, MA, USA) with 300 MHz magnet. Gel permeation chromatography (GPC) was performed to determine *M_n_* and *M_w_*/*M_n_* of PEUG# with reference to standardized polystyrenes using UV detector and THF as eluting reagent. The transmittance of the polymer films on the glass substrates was acquired using ultraviolet-visible (UV-Vis) spectroscopy (Perkin Elmer Lambda 20 spectrometer, PerkinElmer, Inc., Waltham, MA, USA). The contact angles for distilled water and diiodomethane on PEUG# films were detected with contact angle analyzer (Kruss DSA10, KRÜSS scientific instruments Inc., Hamburg, Germany)-installed drop shape analysis software. The surface energy value was measured by the Owens-Wendt’s equation:

(1) $$\gamma_{sl}=\gamma_{s}+\gamma_{l}-2{\left(\gamma_{s}^{d}\gamma_{l}^{d}\right)}^{1/2}-2{\left(\gamma_{s}^{p}\gamma_{l}^{p}\right)}^{1/2}$$

in which *γ_l_* is the surface energy of the liquid, *γ_sl_* is the interfacial energy of the solid/liquid interface, *γ_s_* is the surface energy of the solid, $\gamma_{l}^{d}$ and $\gamma_{l}^{p}$ are known for the test liquids, and $\gamma_{s}^{d}$ and $\gamma_{s}^{p}$ can be calculated from the measured static contact angles [49]. Polarized optical microscopy (POM) images of LC cells were photographed using optical microscopy (Nikon, Eclipse E600 POL, NIKON Co., Tokyo, Japan) installed polarizer and digital camera (Nikon, Coolpix 995, NIKON Co., Tokyo, Japan). Voltage holding ratio (VHR) was determined with VHR measurement system (VHRM 105, Autronic-Melchers, Autronic-Melchers CDT. Ltd., Karlsruhe, Germany) under following conditions (data voltage, pulse width, and frame frequency were 1.0 V, 64 μs, and 60 Hz, respectively). The measuring temperatures were 25 and 60 °C. Residual DC voltage (R-DC) was assessed by capacitance-voltage (C-V) hysteresis method using Nissan Chemical Industries, Ltd. (Tokyo, Japan).

## 3. Results and Discussion

### 3.1. Synthesis and Characterization of Eugenol Modified Polystyrene

**Figure 1:**
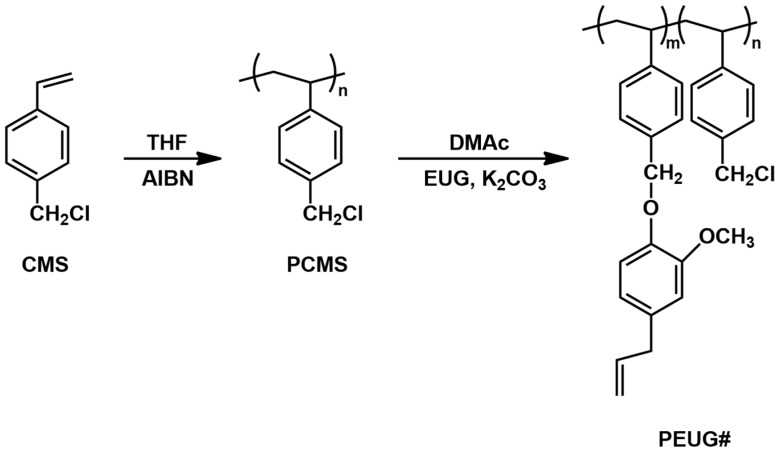
Synthetic route to eugenol modified polystyrene (PEUG#), where # indicates the mole percent of eugenol containing monomeric units in the polymer.

Figure 1 shows the synthetic routes to the PEUG100 and copolymers (PEUG80, PEUG60, PEUG40, and PEUG20). The copolymers that had different degrees (%) of substitution were obtained by varying the amounts of eugenol in the reaction as previously reported similar procedure [48]. Conversions of nearly 100% from chloromethyl to eugenyl methyl ether were obtained when 150 mol % of eugenol was used at 75 °C for 24 h. The chemical constituents of monomeric units in PEUG# were checked by ^1^H NMR spectra. The ^1^H NMR spectrum of PEUG100 shows the existence of protons from styrene backbone (δ = 6.3–7.2 ppm). The proton peaks of eugenol side chains (δ = 3.1–3.2 (2H), 3.7–3.8 (3H), 5.0–5.9 (2H), 5.8–6.0 (1H)) indicate the inclusion of eugenol moieties in the polymer. The content of eugenol was calculated by comparing the integration value of the proton peaks of eugenol (δ = 3.1–3.2 and 3.7–3.8) and the chloromethyl side chains (δ = 4.7–5.0). Similar integrations and calculations were performed for PEUG80, PEUG60, PEUG40, and PEUG20 and were typically within ±5% of the expected values.

The *M_n_* values of the polymer series synthesized from the PCMS (*M_n_* = 30,000) were always larger than 31,000, indicating that the polymer modification from PCMS to the polymers resulted in an increase in the *M_n_* values of the polymers, which was expected result (Table 1). PEUG# series are soluble in many intermediate polar solvents with low boiling points (e.g., chloroform and THF), and in aprotic polar solvents (e.g., *N,N′*-dimethylformamide (DMF), *N,N′*-dimethylacetamide (DMAc), *N*-methyl-2-pyrrolidone (NMP)). The solubility of all samples for various solvents indicated their suitability as thin film materials for flexible devices.

**Figure 2:**
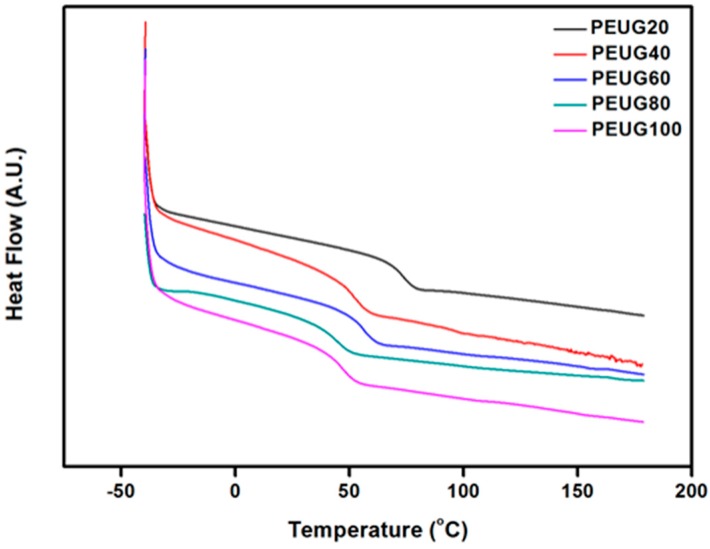
DSC (differential scanning calorimetry) thermogram of eugenol modified polystyrene (PEUG#).

The thermal behaviors of PEUG# were investigated by differential scanning calorimetry (DSC). All PEUG# series were amorphous. This can be explained that only one glass transition was monitored from the DSC thermograms. As the molar ratio of PEUG# side group increased, PEUG20 to PEUG100, *T_g_* decreased from 74 °C to 47 °C (Figure 2). In general, the *T_g_* value of polymers depends on the polarity, flexibility, and bulkiness of the side group. It has been reported that the *T_g_* values of polymers increased with increasing polarity of the side group [50], while the values can be increased or decreased according to an increase of side group bulkiness. For example, the *T_g_* of the poly(vinylnaphthalene) with relatively bulky substituents, such as naphthalene, is higher than that of polystyrene [50]. This means that the incorporation of a bulky side group decreases the flexibility of the polymer backbone. In this case, when the bulky side chain such as eugenol was incorporated, the free volume of the polymer was increased, which increased the distance between the polymer chain segments [51]. Therefore, the intermolecular and intersegmental interactions are weaker, which decreases the *T_g_* value [52,53,54].

### 3.2. Transmittance of Eugenol Modified Polystyrene Film

**Figure 3:**
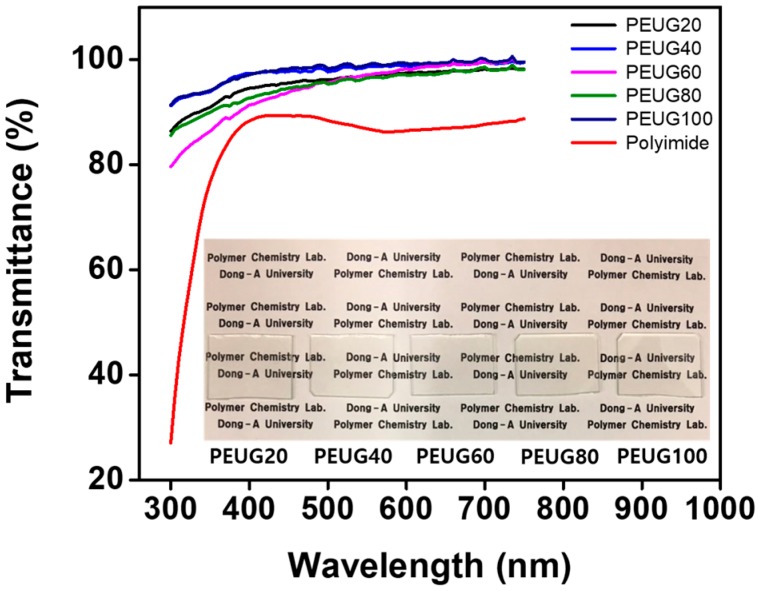
Optical transmittance spectra of eugenol modified polystyrene (PEUG#) and polyimide orientation layers onto quartz substrates.

A quantitative analysis of the transparency of the PEUG# films was performed using UV-Vis spectroscopy to investigate the possibility of surface-coating applications (Figure 3). The transmittance value of the PEUG# film coated onto the glass substrate is in range of 96.5–99.0% at 550 nm, which is higher than the transmittance value (80.5%) of the widely used polyimide film, which has an intrinsic yellowish coloration issue related to the diimide fragment conjugation; this reduces the usability of the polyimide film as an LC orientation layer. The results show that the optical transparency of the PEUG# film in the visible light region is sufficiently good to enable the use of the film as an optical material for display devices.

### 3.3. LC Orientation Behavior of the LC Cells Fabricated with Eugenol Modified Polystyrene Films

**Figure 4:**
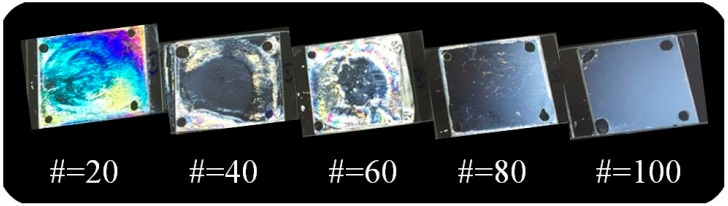
Photograph images of the LC cells made from PEUG# films according to the molar content of eugenol moiety.

Figure 4 shows the photograph images of the LC cells fabricated from the copolymers (PEUG#). The LC cell fabricated using the PEUG# film with the eugenol side group content of 20 mol % (PEUG20) shows a random planar LC orientation, while random planar and/or tilted LC orientations are observed for the LC cells fabricated using the PEUG40 and PEUG60 polymer films. Good uniformity of the vertical LC orientation behavior was observed for the LC cells fabricated with polymer films with a eugenol side group content of more than 80 mol % (PEUG80 and PEUG100). PEUG# (# = 80, and 100) films showed stabilized vertical LC orientation in whole area and lasted for more than several months. Therefore, as the molar ratio of side groups in PEUG# increases, the vertical LC orientation also increases.

**Figure 5:**
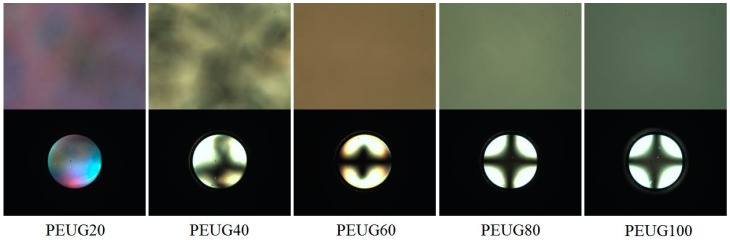
Polarized optical microscopy (POM) images of the LC cells made from PEUG#.

As shown Figure 5, the LC orientation performances on PEUG# film were investigated using POM images. A random planar LC orientation on PCMS film was observed. At a molar content of 20% of the eugenol containing monomeric unit in the PEUG#, the LC cells fabricated with the PEUG# film exhibited planar LC orientation in the conoscopic POM images. At a molar content in the range of 40–60%, the LC cells fabricated with the PEUG# film exhibited a random tilted LC orientation in the conoscopic POM images. On the other hand, all the PEUG80 and PEUG100 films exhibited stable vertical LC orientation layers.

### 3.4. Surface Properties of Eugenol Modified Polystyrene Films

**Figure 6:**
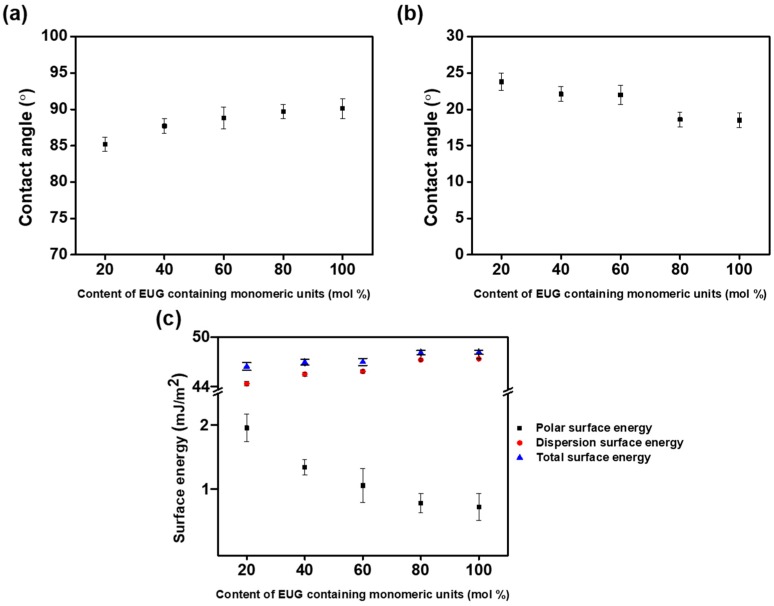
(**a**) Water, (**b**) diiodomethane contact angle, and (**c**) surface energy values of PEUG# films according to the molar content of the eugenol moiety in the side groups.

The results obtained for the LC orientation behavior indicated a general trend of the occurrence of vertical LC orientation with increasing molar content of the eugenol side groups. It is known that high pretilt angles of LC molecules resulting in vertical orientation performance are associated with low surface energy of the orientation layer and/or sterically repulsive force between LC molecules and orientation layers [55,56]. For example, polyimide derivatives that have nonpolar and bulky groups (e.g., pentylcyclohexylbenzene [55] and 4-(*n*-octyloxy)phenyloxy [56]) exhibited vertical orientation behavior. Therefore, we tried to explain the LC orientation performances of the PEUG# films utilizing surface energy value measurements, one of surface characterization techniques. Surface energy values were calculated based on contact angles of (distilled) water and diiodomethane (Figure 6 and Table 2). The total surface energy values were calculated by Owens-Wendt’s equation, which is summation of polar and dispersion terms. The total surface energy values of PEUG# films according to the molar content of the eugenol moiety in the side groups increased to 46.66, 47.14, 47.18, 48.23, and 48.28 mJ m^−2^. The dispersion surface energy values according to the molar content of the eugenol moiety also increased to 44.70, 45.78, 46.10, 47.42, and 47.53 mJ m^−2^. On the other hand, the polar surface energy values of PEUG# films according to the molar content of the eugenol moiety in the side groups decreased to 1.97, 1.36, 1.08, 0.81, and 0.75 mJ m^−2^. We have found that the vertical LC orientation was well correlated with the polar surface energy term of the polymer film. It has been described that the polar surface energy of polymer film can affect the LC orientation behavior, as previously reported by other research [57,58,59]. Since eugenol has a nonpolar and bulky group, such as not only phenyl group but allyl group attached to phenyl group in the *para* position, the increase in the degree of substitution of EUG causes a decrease in the polar surface energy, in which vertical orientation could be formed. Therefore, it is reasonable to conclude that the vertical LC orientation behavior of PEUG100 and PEUG80 is due to the increased steric repulsions between the LC molecules and the polymer surfaces that result from the incorporation of the nonpolar and bulky eugenol moieties into the side group of the PS and the low polar surface energy originating from the unique chemical structure of nonpolar and long carbon groups.

### 3.5. Reliability and Electro-Optical Performance of the LC Cells Fabricated with Eugenol Modified Polystyrene Films

**Figure 7:**
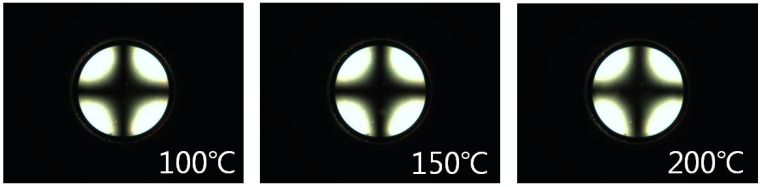
Conoscopic POM images of PEUG100 LC cells, after heat treatment at 100, 150, and 200 °C for 10 min, respectively.

The reliability of the LC cells fabricated from the polymer films was investigated using a stability test of the LC orientation under harsh conditions such as high temperatures. The thermal stability of the LC cell fabricated from the PEUG100 film was estimated from the POM image after heating for 10 min at 100, 150, and 200 °C, respectively. As shown in Figure 7, there are no distinguishable differences in the pretilt angle of the PEUG100 film with vertical LC orientation as shown in the Maltese cross pattern in the conoscopic POM images, indicating that the vertical LC orientation of the PEUG100 LC cell was maintained, even at a high temperature.

The electro-optical (E-O) performance of the LC cell fabricated with the PEUG100 film was measured with regard to potential and practical display applications. Voltage holding ratio (VHR) of LC cell was demonstrated more than 99% at 25 °C, which was maintained at 60 °C. The VHR is enough to be high for substantive applications as the LC orientation layer in thin film transistor (TFT)-addressed display [11]. The R-DC measured by C-V hysteresis method of the LC cell was discovered to be very low, about 10 mV, which is even smaller than the R-DC of polyimides [11]. The excellent thermal stability, VHR, and R-DC of the LC cell fabricated using the PEUG100 film was ascribed to the intrinsic properties of polymers with high carbon contents, such as good thermal stability and a low dielectric constant. 

Recently, considerable efforts have been devoted to the development of plastic substrates for flexible displays [60]. All the LC cells fabricated from the PEUG# films on the polyethylene terephthalate (PET) substrates exhibited similar LC orientation behavior compared with LC cells fabricated from the same polymer films on glass substrates (Figure 4 and Figure 5). We found that the LC cells fabricated using the PEUG100 film on plastic PET substrates showed vertical LC orientation behavior. Furthermore, this type of LC cell exhibited a very good vertical LC orientation that was maintained after bending it several hundred times. Therefore, as a plant-based and renewable resource, the PEUG100 film can be considered a potential material for LC orientation layers for eco-friendly flexible displays.

## 4. Conclusions

A series of PS derivatives containing plant-based and renewable eugenol side groups (PEUG#) were synthesized to determine the LC orientation properties of the polymer films. The LC cells fabricated from the polymer films with more than 80 mol % of eugenol (PEUG80 and PEUG100) exhibited vertical LC orientation. However, LC cells made using PEUG# films with less than 60 mol % of eugenol exhibited random planar and/or tilted LC orientation behavior. The PEUG100 polymer films exhibited good optical transparency in the visible light region (400–700 nm). At 550 nm, the transmittance value was higher for the PEUG100 film (99.0%) than for the polyimide film (80.5%), the most commonly used LC orientation layer. The vertical LC orientation was ascribed to the steric repulsions between the LC molecules and the polymer surfaces due to the incorporation of a nonpolar and bulky eugenol moiety into the side chain. In addition, the vertical LC orientation was well correlated with the polar surface energy values of the polymer induced by the long alkyl groups. These results provide a basis for the design of eco-friendly and renewable LC orientation layers based on eugenol containing polymer films.

## Figures and Tables

**Table 1:** Reaction conditions and characterizations for the synthesis of the PCMS and PEUG#.

Polymer Designation	Eugenol [mol %]	Degree of Substitution [%]	*M_n_* ^1^	*M_w_*/*M_n_* ^1^
PCMS	-	-	3000	2.10
PEUG20	20	20	31,000	2.22
PEUG40	40	40	34,000	2.54
PEUG60	60	60	37,000	2.69
PEUG80	80	80	37,000	2.55
PEUG100	150	100	38,000	2.48

^1^ Obtained from GPC using tetrahydrofuran as solvent with respect to monodisperse polystyrene as standard.

**Table 2:** Contact angle and surface energy values and LC orientation properties on the polymer films.

Polymer Designation	Contact Angle [o] ^1^		Surface Energy [mJ/m^2^] ^2^			LC Aligning Ability ^3^
	Water	Diiodomethane	Polar	Dispersion	Total	
PCMS	71.1	35.2	8.67	37.00	45.67	X
PEUG20	85.2	23.8	1.97	44.70	46.66	X
PEUG40	87.7	22.1	1.36	45.78	47.14	X
PEUG60	88.8	22.0	1.08	46.10	47.18	X
PEUG80	89.7	18.6	0.81	47.42	48.23	O
PEUG100	90.1	18.5	0.75	47.53	48.28	O

^1^ Measured from static contact angles; ^2^ calculated from Owens-Wendt’s equation; ^3^ circle (O) and cross (X) indicate polymer film have vertical and random planar, tilted LC aligning ability, respectively.
